# Correction to “Genome‐wide association study identifies quantitative trait loci associated with resistance to *Verticillium dahliae* race 3 in tomato”

**DOI:** 10.1002/tpg2.70162

**Published:** 2025-11-16

Adhikari, T. B., Olukolu, B. A., Pandey, A., Philbrick, A. N., Panthee, D. R., Shekasteband, R., Gardner, R. G., Dean, R. A., & Louws, F. J. (2025). Genome‐wide association study identifies quantitative trait loci associated with resistance to *Verticillium dahliae* race 3 in tomato. *The Plant Genome*, *18*, e70132. https://doi.org/10.1002/tpg2.70132

The first sentence in the caption of Figure 6 contained an error in mislabeling parts A and B: “The genomic predictive abilities (PA) for LC (A) and CN_perc (B) were computed using genomic best linear unbiased prediction (GBLUP) and GWAS‐assisted best linear unbiased prediction (GWABLUP)” has been corrected to “The genomic predictive abilities (PA) for CN_perc (A) and LC (B) were computed using genomic best linear unbiased prediction (GBLUP) and GWAS‐assisted best linear unbiased prediction (GWABLUP).”

**FIGURE 6:**
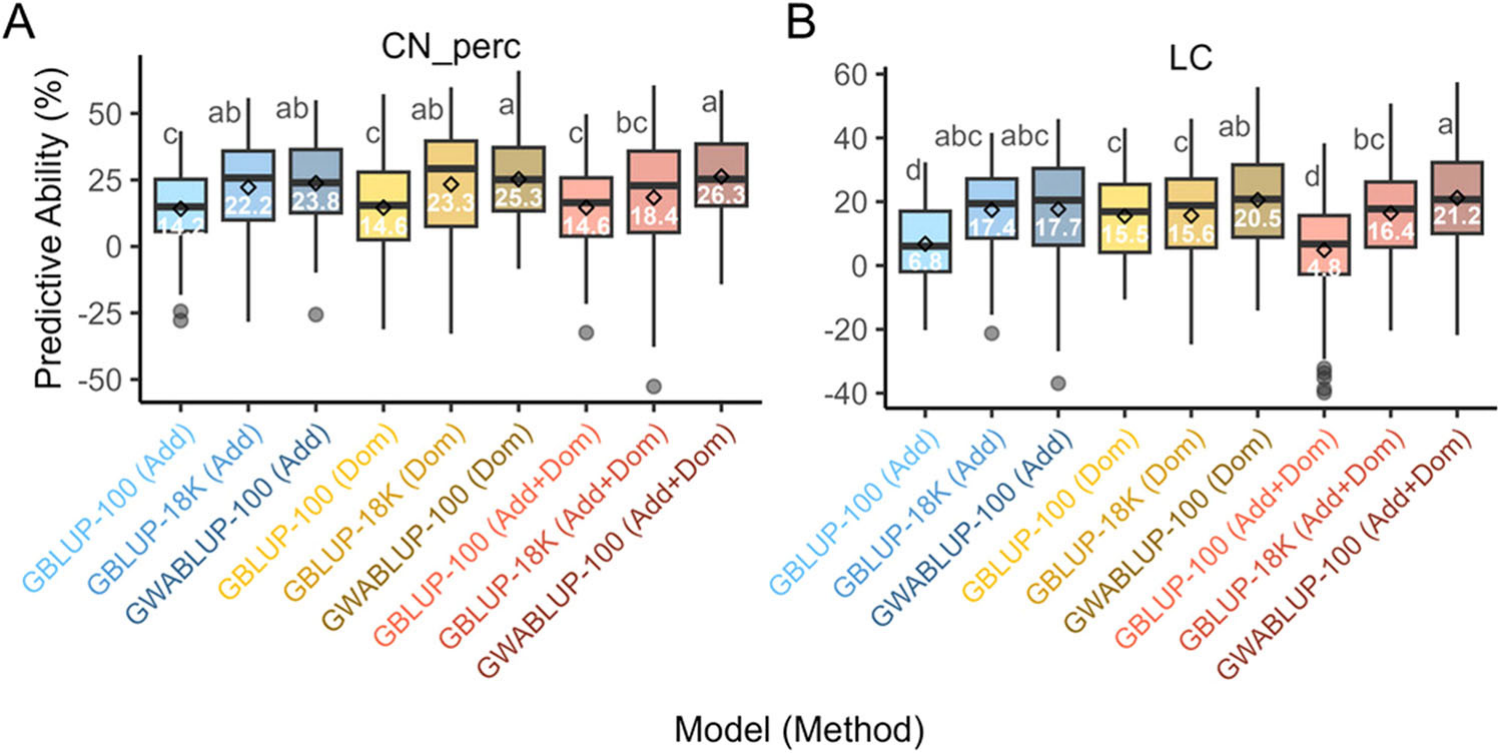
The genomic predictive abilities (PA) for CN_perc (A) and LC (B) were computed using genomic best linear unbiased prediction (GBLUP) and GWAS‐assisted best linear unbiased prediction (GWABLUP). This analysis modeled additive (Add), dominance (Dom), and both additive and dominance (Add + Dom) effects. For marker selection, the top 100 genome‐wide association study (GWAS) hits were identified from each of the 100 iterations of GWAS and model selection, and the final top 100 GWAS hits were based on markers with the highest resample model inclusion probability (RMIP) values. In the box plots, the white text inside the boxes indicates the mean PA values, while the letters from the Duncan multiple range test (DMRT) show the significance of differences between the means. The GBLUP model is based on 100 randomly selected and 18,503 genome‐wide markers.

We apologize for this error.

